# Supplementary material for: CtBP impedes JNK- and Upd/STAT-driven cell fate misspecifications in regenerating Drosophila imaginal discs
Source: eLife. 2018 Jan 26;7:e30391. doi: 10.7554/eLife.30391 (PMC5823544; doi:10.7554/eLife.30391)
Supplement: Supplementary file 1. — The frequency of ectopic wings (EW) following rnts>egr damage for third-chromosome deletions screened. The deletions are ordered based on the cytogenetic position along the third chromosome. [file elife-30391-supp1.docx]

**Supplemental File 1. Screen summary for 3^rd^ chromosome deletions.**

|  | **Bloominton** | **Deletion Name** | **Cytogenetic Position** | **Total Flies** | **with EW(s)** | **EW frequency** | **Notes** |
| --- | --- | --- | --- | --- | --- | --- | --- |
| 1 | 24627 | *Df(3L)ED50002* | 61A1;61B1 | 43 | 1 | 0.023255814 |  |
| 2 | 8047 | *Df(3L)ED201* | 61B1;61C1 | 90 | 0 | 0 |  |
| 3 | 27370 | *Df(3L)BSC798* | 61C1;61C8 | 62 | 1 | 0.016129032 |  |
| 4 | 7564 | *Df(3L)Exel6085* | 61C3;61C9 | 113 | 0 | 0 |  |
| 5 | 8050 | *Df(3L)ED4196* | 61C7;62A2 | 241 | 75 | 0.31120332 |  |
| 6 | 8051 | *Df(3L)ED202* | 61C9;61F7 | 146 | 0 | 0 |  |
| 7 | 23674 | *Df(3L)BSC289* | 61F6;62A9 | 159 | 88 | 0.553459119 |  |
| 8 | 9609 | *Df(3L)BSC178* | 61F8;62A3 | 49 | 1 | 0.020408163 |  |
| 9 | 9693 | *Df(3L)BSC181* | 62A11;62B7 | 99 | 1 | 0.01010101 |  |
| 10 | 7566 | *Df(3L)Exel6087* | 62A2;62A6 | 179 | 145 | 0.810055866 |  |
| 11 | 26522 | *Df(3L)BSC670* | 62A3;62A9 | 71 | 5 | 0.070422535 |  |
| 12 | 8096 | *Df(3L)ED4287* | 62B4;62E5 | 70 | 1 | 0.014285714 |  |
| 13 | 8976 | *Df(3L)BSC119* | 62E7;62F5 | 74 | 0 | 0 |  |
| 14 | 7571 | *Df(3L)Exel6092* | 62F5;63A3 | 65 | 1 | 0.015384615 |  |
| 15 | 26523 | *Df(3L)BSC671* | 63A2;63B11 | 44 | 0 | 0 |  |
| 16 | 8059 | *Df(3L)ED208* | 63C1;63F5 | 101 | 0 | 0 |  |
| 17 | 24392 | *Df(3L)BSC368* | 63F1;64A4 | 136 | 29 | 0.213235294 |  |
| 18 | 7577 | *Df(3L)Exel6098* | 63F2;63F7 | 169 | 1 | 0.00591716 |  |
| 19 | 8060 | *Df(3L)ED4341* | 63F6;64B9 | 128 | 0 | 0 |  |
| 20 | 8061 | *Df(3L)ED210* | 64B9;64C13 | 136 | 0 | 0 |  |
| 21 | 24395 | *Df(3L)BSC371* | 64C1;64E1 | 41 | 3 | 0.073170732 |  |
| 22 | 30589 | *Df(3L)BSC884* | 64D6;64E7 | 84 | 0 | 0 |  |
| 23 | 24914 | *Df(3L)BSC410* | 64E7;65B3 | 94 | 0 | 0 |  |
| 24 | 24915 | *Df(3L)BSC411* | 65A2;65C1 | 0 | 0 | Lethal | **Lethal** |
| 25 | 7588 | *Df(3L)Exel6109* | 65C3;65D3 | 98 | 6 | 0.06122449 |  |
| 26 | 9701 | *Df(3L)BSC224* | 65D5;65E6 | 93 | 0 | 0 |  |
| 27 | 8974 | *Df(3L)BSC117* | 65E9;65F5 | 51 | 0 | 0 |  |
| 28 | 30580 | *Df(3L)BSC875* | 65F5;66A8 | 99 | 0 | 0 |  |
| 29 | 27575 | *Df(3L)BSC807* | 66A8;66B12 | 72 | 0 | 0 |  |
| 30 | 24400 | *Df(3L)BSC376* | 66B8;66C8 | 147 | 0 | 0 |  |
| 31 | 24413 | *Df(3L)BSC389* | 66C12;66D8 | 70 | 0 | 0 |  |
| 32 | 27576 | *Df(3L)BSC815* | 66C3;66D4 | 87 | 0 | 0 |  |
| 33 | 25687 | *Df(3L)BSC612* | 66D10;66E1 | 42 | 2 | 0.047619048 |  |
| 34 | 8066 | *Df(3L)ED4421* | 66D12;67B3 | 78 | 0 | 0 |  |
| 35 | 27577 | *Df(3L)BSC816* | 66D9;66D12 | 275 | 24 | 0.087272727 |  |
| 36 | 997 | *Df(3L)AC1* | 67A2;67D11--13 | 93 | 0 | 0 |  |
| 37 | 8970 | *Df(3L)BSC113* | 67B1;67B5 | 151 | 7 | 0.046357616 |  |
| 38 | 24415 | *Df(3L)BSC391* | 67B7;67C5 | 46 | 0 | 0 |  |
| 39 | 24416 | *Df(3L)BSC392* | 67C4;67D1 | 110 | 4 | 0.036363636 |  |
| 40 | 26525 | *Df(3L)BSC673* | 67C7;67D10 | 113 | 0 | 0 |  |
| 41 | 9355 | *Df(3L)ED4457* | 67E2;68A7 | 87 | 14 | 0.16091954 |  |
| 42 | 24943 | *Df(3L)BSC439* | 67E4;68A1 | 249 | 2 | 0.008032129 |  |
| 43 | 27578 | *Df(3L)BSC817* | 68A4;68B4 | 235 | 11 | 0.046808511 |  |
| 44 | 8068 | *Df(3L)ED4470* | 68A6;68E1 | 130 | 24 | 0.184615385 |  |
| 45 | 24403 | *Df(3L)BSC379* | 68B1;68C10 | 239 | 0 | 0 |  |
| 46 | 8069 | *Df(3L)ED4475* | 68C13;69B4 | 126 | 14 | 0.111111111 |  |
| 47 | 8070 | *Df(3L)ED4483* | 69A5;69D3 | 143 | 0 | 0 |  |
| 48 | 8072 | *Df(3L)ED4486* | 69C4;69F6 | 117 | 6 | 0.051282051 |  |
| 49 | 8097 | *Df(3L)ED4502* | 70A3;70C10 | 158 | 1 | 0.006329114 |  |
| 50 | 8073 | *Df(3L)ED4543* | 70C6;70F4 | 113 | 0 | 0 |  |
| 51 | 8074 | *Df(3L)ED217* | 70F4;71E1 | 112 | 3 | 0.026785714 |  |
| 52 | 24945 | *Df(3L)BSC441* | 71A1;71B4 | 156 | 2 | 0.012820513 |  |
| 53 | 27916 | *Df(3L)BSC837* | 71A4;71B6 | 175 | 0 | 0 |  |
| 54 | 8075 | *Df(3L)ED218* | 71B1;71E1 | 144 | 2 | 0.013888889 |  |
| 55 | 24946 | *Df(3L)BSC442* | 71D2;71E3 | 104 | 3 | 0.028846154 |  |
| 56 | 25120 | *Df(3L)BSC558* | 71D3;71E1 | 342 | 4 | 0.011695906 |  |
| 57 | 27888 | *Df(3L)BSC845* | 71D3;72A1 | 369 | 33 | 0.089430894 |  |
| 58 | 27914 | *Df(3L)BSC833* | 71D4;71F1 | 164 | 5 | 0.030487805 |  |
| 59 | 27587 | *Df(3L)BSC575* | 71F1;72C1 | 135 | 1 | 0.007407407 |  |
| 60 | 27346 | *Df(3L)BSC774* | 71F1;72D10 | 86 | 0 | 0 |  |
| 61 | 24947 | *Df(3L)BSC443* | 72B1;72E4 | 30 | 0 | 0 |  |
| 62 | 29021 | *Df(3L)BSC831* | 72C1;72C1 | 79 | 0 | 0 |  |
| 63 | 8078 | *Df(3L)ED4606* | 72D4;73C4 | 67 | 1 | 0.014925373 |  |
| 64 | 25117 | *Df(3L)BSC555* | 72E2;73A10 | 89 | 0 | 0 |  |
| 65 | 8098 | *Df(3L)ED4674* | 73B5;73E5 | 163 | 1 | 0.006134969 |  |
| 66 | 8099 | *Df(3L)ED4685* | 73D5;74E2 | 42 | 0 | 0 |  |
| 67 | 8100 | *Df(3L)ED4710* | 74D1;75B11 | 67 | 0 | 0 |  |
| 68 | 27347 | *Df(3L)BSC775* | 75A2;75E4 | 80 | 3 | 0.0375 |  |
| 69 | 9697 | *Df(3L)BSC220* | 75F1;76A1 | 159 | 0 | 0 |  |
| 70 | 8087 | *Df(3L)ED229* | 76A1;76E1 | 75 | 0 | 0 |  |
| 71 | 8088 | *Df(3L)ED4858* | 76D3;77C1 | 182 | 19 | 0.104395604 |  |
| 72 | 27917 | *Df(3L)BSC839* | 77B4;77C6 | 94 | 0 | 0 |  |
| 73 | 27369 | *Df(3L)BSC797* | 77C3;78A1 | 168 | 0 | 0 |  |
| 74 | 24953 | *Df(3L)BSC449* | 77F2;78C2 | 54 | 0 | 0 |  |
| 75 | 25116 | *Df(3L)BSC553* | 78A2;78C2 | 51 | 0 | 0 |  |
| 76 | 24923 | *Df(3L)BSC419* | 78C2;78D8 | 251 | 46 | 0.183266932 |  |
| 77 | 24939 | *Df(3L)BSC435* | 78C6;78F1 | 306 | 13 | 0.04248366 |  |
| 78 | 24922 | *Df(3L)BSC418* | 78C9;78E1 | 87 | 54 | 0.620689655 |  |
| 79 | 7950 | *Df(3L)Exel9066* | 78D5;78D6 | 155 | 0 | 0 |  |
| 80 | 8101 | *Df(3L)ED4978* | 78D5;79A2 | 692 | 77 | 0.111271676 |  |
| 81 | 23669 | *Df(3L)BSC284* | 78F1;79B1 | 149 | 3 | 0.020134228 |  |
| 82 | 9700 | *Df(3L)BSC223* | 79A3;79B3 | 147 | 0 | 0 |  |
| 83 | 23149 | *Df(3L)BSC249* | 79B2;79D1 | 299 | 52 | 0.173913043 |  |
| 84 | 24955 | *Df(3L)BSC451* | 79B2;79F5 | 136 | 20 | 0.147058824 |  |
| 85 | 8089 | *Df(3L)ED230* | 79C2;80A4 | 156 | 0 | 0 |  |
| 86 | 8102 | *Df(3L)ED5017* | 80A4;80C2 | 140 | 2 | 0.014285714 |  |
| 87 | 7002 | *Df(3L)1-16* | 80F;80F | 203 | 4 | 0.019704433 |  |
| 88 | 2596 | *Df(3L)6B-29* | 80Fd;80Fj | 202 | 0 | 0 |  |
| 89 | 9196 | *Df(3R)ED5021* | 81F6;82A5 | 100 | 9 | 0.09 |  |
| 90 | 9197 | *Df(3R)ED5046* | 81F6;82D2 | 40 | 9 | 0.225 |  |
| 91 | 9226 | *Df(3R)ED5100* | 81F6;82E7 | 31 | 1 | 0.032258065 |  |
| 92 | 2597 | *Df(3R)10-65* | 81Fa;81Fa | 192 | 5 | 0.026041667 |  |
| 93 | 7619 | *Df(3R)Exel6140* | 82A1;82A4 | 83 | 2 | 0.024096386 |  |
| 94 | 8967 | *Df(3R)ED5147* | 82E7;83A1 | 176 | 0 | 0 |  |
| 95 | 8965 | *Df(3R)ED5156* | 82F8;83A4 | 127 | 1 | 0.007874016 |  |
| 96 | 7623 | *Df(3R)Exel6144* | 83A6;83B6 | 106 | 2 | 0.018867925 |  |
| 97 | 25077 | *Df(3R)BSC549* | 83A6;83B6 | 165 | 11 | 0.066666667 |  |
| 98 | 7443 | *Df(3R)BSC47* | 83B7--C1;83C6--D1 | 157 | 1 | 0.006369427 |  |
| 99 | 24968 | *Df(3R)BSC464* | 83B7;83E1 | 194 | 2 | 0.010309278 |  |
| 100 | 26533 | *Df(3R)BSC681* | 83E2;83E5 | 66 | 2 | 0.03030303 |  |
| 101 | 9620 | *Df(3R)BSC193* | 83E5;83F4 | 200 | 0 | 0 |  |
| 102 | 26836 | *Df(3R)BSC738* | 83E5;84A1 | 185 | 0 | 0 |  |
| 103 | 24971 | *Df(3R)BSC467* | 83F1;84B2 | 107 | 1 | 0.009345794 |  |
| 104 | 1842 | *Df(3R)Antp17* | 84A5;84D9 | 136 | 0 | 0 |  |
| 105 | 25724 | *Df(3R)BSC633* | 84B2;84C3 | 133 | 0 | 0 |  |
| 106 | 8685 | *Df(3R)ED7665* | 84B4;84E11 | 7 | 0 | 0 | **Sub-Viable** |
| 107 | 9076 | *Df(3R)ED5223* | 84D9;84E11 | 8 | 2 | 0.25 | **Sub-Viable** |
| 108 | 25017 | *Df(3R)BSC513* | 84D9;84F6 | 18 | 3 | 0.166666667 |  |
| 109 | 8682 | *Df(3R)ED5230* | 84E6;85A5 | 77 | 14 | 0.181818182 |  |
| 110 | 9338 | *Df(3R)ED5296* | 84F6;85C3 | 78 | 0 | 0 |  |
| 111 | 9077 | *Df(3R)ED5330* | 85A5;85D1 | 19 | 0 | 0 |  |
| 112 | 25010 | *Df(3R)BSC506* | 85B1;85C2 | 122 | 0 | 0 |  |
| 113 | 26518 | *Df(3R)BSC666* | 85C2;85D11 | 106 | 19 | 0.179245283 |  |
| 114 | 9203 | *Df(3R)ED5331* | 85C3;85D1 | 136 | 0 | 0 |  |
| 115 | 9204 | *Df(3R)ED5339* | 85D1;85D11 | 170 | 40 | 0.235294118 |  |
| 116 | 7955 | *Df(3R)Exel9036* | 85D11;85D11 | 149 | 0 | 0 |  |
| 117 | 24980 | *Df(3R)BSC476* | 85D16;85D24 | 106 | 0 | 0 |  |
| 118 | 7731 | *Df(3R)Exel6264* | 85D24;85E5 | 23 | 0 | 0 |  |
| 119 | 25011 | *Df(3R)BSC507* | 85D6;85D15 | 242 | 122 | 0.504132231 |  |
| 120 | 9227 | *Df(3R)ED5428* | 85E1;85F8 | 259 | 5 | 0.019305019 |  |
| 121 | 25054 | *Df(3R)BSC526* | 85E8;85F14 | 214 | 0 | 0 |  |
| 122 | 7633 | *Df(3R)Exel6154* | 85E9;85F1 | 82 | 0 | 0 |  |
| 123 | 9082 | *Df(3R)ED5474* | 85F11;86B1 | 162 | 3 | 0.018518519 |  |
| 124 | 9215 | *Df(3R)ED5495* | 85F16;86C7 | 279 | 0 | 0 |  |
| 125 | 24983 | *Df(3R)BSC479* | 86A3;86C7 | 216 | 1 | 0.00462963 |  |
| 126 | 25126 | *Df(3R)BSC568* | 86C7;86D7 | 176 | 2 | 0.011363636 |  |
| 127 | 9084 | *Df(3R)ED5518* | 86C7;86E13 | 162 | 1 | 0.00617284 |  |
| 128 | 24973 | *Df(3R)BSC469* | 86D8;87A2 | 104 | 0 | 0 |  |
| 129 | 8920 | *Df(3R)ED5559* | 86E11;87B11 | 93 | 6 | 0.064516129 |  |
| 130 | 8029 | *Df(3R)ED5577* | 86F9;87B13 | 136 | 0 | 0 |  |
| 131 | 25018 | *Df(3R)BSC514* | 86F9;87B2 | 96 | 1 | 0.010416667 |  |
| 132 | 7642 | *Df(3R)Exel6163* | 87A1;87A4 | 102 | 1 | 0.009803922 |  |
| 133 | 3007 | *Df(3R)ry615* | 87B10-11;87E7-8 | 18 | 0 | 0 |  |
| 134 | 24990 | *Df(3R)BSC486* | 87B10;87E9 | 221 | 7 | 0.031674208 |  |
| 135 | 9087 | *Df(3R)ED5610* | 87B11;87D7 | 67 | 1 | 0.014925373 |  |
| 136 | 9206 | *Df(3R)ED5573* | 87B5;87B13 | 140 | 0 | 0 |  |
| 137 | 7972 | *Df(3R)Exel7318* | 87C7;87D5--6 | 78 | 2 | 0.025641026 |  |
| 138 | 9088 | *Df(3R)ED5612* | 87C7;87F6 | 231 | 37 | 0.16017316 |  |
| 139 | 7646 | *Df(3R)Exel6167* | 87D10;87E3 | 109 | 2 | 0.018348624 |  |
| 140 | 25690 | *Df(3R)BSC615* | 87D4;87E3 | 103 | 15 | 0.145631068 |  |
| 141 | 7973 | *Df(3R)Exel8157* | 87D8;87D10 | 412 | 196 | 0.475728155 |  |
| 142 | 7974 | *Df(3R)Exel8158* | 87E3;87E8 | 87 | 0 | 0 |  |
| 143 | 37537 | *Df(3R)ED5623* | 87E3;88A4 | 35 | 0 | 0 |  |
| 144 | 8959 | *Df(3R)ED5622* | 87F10;88A4 | 112 | 0 | 0 |  |
| 145 | 9279 | *Df(3R)ED5642* | 87F10;88C2 | 66 | 7 | 0.106060606 |  |
| 146 | 25444 | *Df(3R)BSC611* | 87F13;88A4 | 18 | 0 | 0 |  |
| 147 | 7648 | *Df(3R)Exel6169* | 87F2;87F10 | 235 | 0 | 0 |  |
| 148 | 9090 | *Df(3R)ED5644* | 88A4;88C9 | 105 | 1 | 0.00952381 |  |
| 149 | 24991 | *Df(3R)BSC487* | 88B1;88B5 | 155 | 1 | 0.006451613 |  |
| 150 | 23714 | *Df(3R)ED10555* | 88C9;88D8 | 155 | 1 | 0.006451613 |  |
| 151 | 24137 | *Df(3R)ED5664* | 88D1;88E3 | 70 | 13 | 0.185714286 |  |
| 152 | 9152 | *Df(3R)ED5705* | 88E12;89A5 | 180 | 26 | 0.144444444 |  |
| 153 | 24138 | *Df(3R)ED10566* | 88E2;88E5 | 145 | 0 | 0 |  |
| 154 | 26839 | *Df(3R)BSC741* | 88E8;88F1 | 136 | 1 | 0.007352941 |  |
| 155 | 25019 | *Df(3R)BSC515* | 88F6;89A8 | 37 | 0 | 0 |  |
| 156 | 7983 | *Df(3R)Exel7328* | 89A12;89B6 | 207 | 8 | 0.038647343 |  |
| 157 | 7982 | *Df(3R)Exel7327* | 89A8;89B1 | 196 | 0 | 0 |  |
| 158 | 7736 | *Df(3R)Exel6269* | 89B12;89B18 | 155 | 0 | 0 |  |
| 159 | 7985 | *Df(3R)Exel7330* | 89B13;89B17 | 171 | 0 | 0 |  |
| 160 | 7737 | *Df(3R)Exel6270* | 89B18;89D8 | 78 | 0 | 0 |  |
| 161 | 30592 | *Df(3R)BSC887* | 89B6;89B16 | 162 | 71 | 0.438271605 |  |
| 162 | 9481 | *Df(3R)ED10639* | 89B7;89B18 | 69 | 0 | 0 |  |
| 163 | 7984 | *Df(3R)Exel7329* | 89B9;89B13 | 182 | 69 | 0.379120879 |  |
| 164 | 57340 | *Df(3R)bor-asun[d93]* | 89B9;89B9 | 181 | 12 | 0.066298343 |  |
| 165 | 8104 | *Df(3R)ED5780* | 89E11;90C1 | 91 | 18 | 0.197802198 |  |
| 166 | 25388 | *Df(3R)BSC564* | 89E13;90B7 | 42 | 8 | 0.19047619 |  |
| 167 | 26846 | *Df(3R)BSC748* | 89E5;89E11 | 105 | 0 | 0 |  |
| 168 | 26826 | *Df(3R)BSC565* | 90A2;90D1 | 95 | 5 | 0.052631579 |  |
| 169 | 25740 | *Df(3R)BSC650* | 90C6;91A2 | 124 | 0 | 0 |  |
| 170 | 9208 | *Df(3R)ED5815* | 90F4;91B8 | 197 | 0 | 0 |  |
| 171 | 25013 | *Df(3R)BSC509* | 91A3;91D5 | 71 | 0 | 0 |  |
| 172 | 7659 | *Df(3R)Exel6180* | 91B5;91C5 | 75 | 1 | 0.013333333 |  |
| 173 | 26840 | *Df(3R)BSC742* | 91B8;91F1 | 137 | 3 | 0.02189781 |  |
| 174 | 24139 | *Df(3R)ED5938* | 91D4;92A11 | 110 | 0 | 0 |  |
| 175 | 27379 | *Df(3R)BSC808* | 92A11;92E1 | 125 | 1 | 0.008 |  |
| 176 | 27380 | *Df(3R)BSC809* | 92A3;92B3 | 142 | 4 | 0.028169014 |  |
| 177 | 25020 | *Df(3R)BSC516* | 92C6;92F13 | 169 | 4 | 0.023668639 |  |
| 178 | 7664 | *Df(3R)Exel6185* | 92E2;92F1 | 70 | 0 | 0 |  |
| 179 | 25022 | *Df(3R)BSC518* | 92E8;92F13 | 159 | 4 | 0.025157233 |  |
| 180 | 9289 | *Df(3R)BSC124* | 92F10;92F13 | 111 | 4 | 0.036036036 |  |
| 181 | 24992 | *Df(3R)BSC488* | 92F2;92F13 | 196 | 33 | 0.168367347 |  |
| 182 | 9501 | *Df(3R)BSC141* | 92F2;93A1 | 341 | 215 | 0.630498534 |  |
| 183 | 27580 | *Df(3R)BSC819* | 93A2;93B8 | 79 | 1 | 0.012658228 |  |
| 184 | 9487 | *Df(3R)ED10845* | 93B9;93D4 | 86 | 3 | 0.034883721 |  |
| 185 | 26529 | *Df(3R)BSC677* | 93D1;93F14 | 24 | 0 | 0 |  |
| 186 | 8923 | *Df(3R)ED6085* | 93F14;94B5 | 167 | 17 | 0.101796407 |  |
| 187 | 8684 | *Df(3R)ED6096* | 94B5;94E7 | 113 | 6 | 0.053097345 |  |
| 188 | 25694 | *Df(3R)BSC619* | 94D10;94E13 | 269 | 72 | 0.267657993 |  |
| 189 | 27375 | *Df(3R)BSC803* | 94D9;94E8 | 116 | 10 | 0.086206897 |  |
| 190 | 7741 | *Df(3R)Exel6274* | 94E4;94E11 | 104 | 1 | 0.009615385 |  |
| 191 | 7990 | *Df(3R)Exel9012* | 94E9;94E13 | 116 | 0 | 0 |  |
| 192 | 9497 | *Df(3R)BSC137* | 94F1;95A4 | 85 | 0 | 0 |  |
| 193 | 24993 | *Df(3R)BSC489* | 94F3;95D1 | 158 | 15 | 0.094936709 |  |
| 194 | 7675 | *Df(3R)Exel6196* | 95C12;95D8 | 151 | 12 | 0.079470199 |  |
| 195 | 9347 | *Df(3R)ED6187* | 95D10;96A7 | 262 | 70 | 0.267175573 |  |
| 196 | 7676 | *Df(3R)Exel6197* | 95D8;95E1 | 131 | 57 | 0.435114504 |  |
| 197 | 24996 | *Df(3R)BSC492* | 95E7;96B17 | 106 | 4 | 0.037735849 |  |
| 198 | 24965 | *Df(3R)BSC461* | 96B15;96D1 | 151 | 0 | 0 |  |
| 199 | 7681 | *Df(3R)Exel6202* | 96D1;96D1 | 48 | 1 | 0.020833333 |  |
| 200 | 7682 | *Df(3R)Exel6203* | 96E2;96E6 | 150 | 2 | 0.013333333 |  |
| 201 | 7682 | *Df(3R)Exel6203* | 96E2;96E6 | 156 | 2 | 0.012820513 |  |
| 202 | 24909 | *Df(3R)BSC321* | 96E6;96E9 | 121 | 10 | 0.082644628 |  |
| 203 | 9500 | *Df(3R)BSC140* | 96F1;96F10 | 282 | 0 | 0 |  |
| 204 | 24999 | *Df(3R)BSC495* | 96F6;97B4 | 120 | 0 | 0 |  |
| 205 | 25000 | *Df(3R)BSC496* | 97A6;97D4 | 101 | 1 | 0.00990099 |  |
| 206 | 25052 | *Df(3R)BSC524* | 97C3;97D11 | 181 | 3 | 0.016574586 |  |
| 207 | 9210 | *Df(3R)ED6255* | 97D2;97F1 | 199 | 0 | 0 |  |
| 208 | 25001 | *Df(3R)BSC497* | 97E6;98B5 | 35 | 5 | 0.142857143 |  |
| 209 | 24964 | *Df(3R)BSC460* | 98B6;98D2 | 45 | 4 | 0.088888889 |  |
| 210 | 25390 | *Df(3R)BSC567* | 98B6;98E5 | 0 | 0 | Lethal | **Lethal** |
| 211 | 30586 | *Df(3R)BSC881* | 98B8;98D6 | 58 | 0 | 0 |  |
| 212 | 7726 | *Df(3R)Exel6259* | 98C4;98D6 | 61 | 0 | 0 |  |
| 213 | 29997 | *Df(3R)BSC874* | 98E1;99A1 | 37 | 6 | 0.162162162 |  |
| 214 | 25004 | *Df(3R)BSC500* | 98F10;99B7 | 39 | 3 | 0.076923077 |  |
| 215 | 27919 | *Df(3R)BSC846* | 99A1;99B10 | 104 | 18 | 0.173076923 |  |
| 216 | 8925 | *Df(3R)ED6316* | 99A5;99C1 | 112 | 11 | 0.098214286 |  |
| 217 | 25075 | *Df(3R)BSC547* | 99B5;99C2 | 87 | 11 | 0.126436782 |  |
| 218 | 25695 | *Df(3R)BSC620* | 99C5;99D3 | 132 | 0 | 0 |  |
| 219 | 7692 | *Df(3R)Exel6214* | 99D5;99E2 | 82 | 2 | 0.024390244 |  |
| 220 | 25007 | *Df(3R)BSC503* | 99E3;99F6 | 117 | 2 | 0.017094017 |  |
| 221 | 25008 | *Df(3R)BSC504* | 99F4;100A2 | 83 | 0 | 0 |  |
| 222 | 7997 | *Df(3R)Exel7378* | 99F8;100A5 | 127 | 6 | 0.047244094 |  |
| 223 | 24142 | *Df(3R)ED6346* | 100A5;100B1 | 109 | 8 | 0.073394495 |  |
| 224 | 26847 | *Df(3R)BSC749* | 100B1;100C1 | 131 | 0 | 0 |  |
| 225 | 27365 | *Df(3R)BSC793* | 100B5;100C4 | 109 | 4 | 0.036697248 |  |
| 226 | 24143 | *Df(3R)ED6361* | 100C7;100E3 | 191 | 4 | 0.020942408 |  |
